# Adenocarcinoma cells isolated from patients in the presence of cerium and transferrin in vitro

**Published:** 2015

**Authors:** A Zende-Del, MR Gholami, F Abdollahpour, H Ahmadvand

**Affiliations:** * Department of Internal Medicine, Lorestan University of Medical Sciences, Khorramabad, Iran; ** Department of Anatomy, Lorestan University of Medical Sciences, Khorramabad, Iran; *** Department of Biochemistry, Lorestan University of Medical Sciences, Khorramabad, Iran

**Keywords:** adenocarcinoma, cytotoxicity, transferrin, Cerium

## Abstract

**Aim:** Cerium as a trace element in the periodic table is a member of the lanthanide group. Cerium ionic radius and its binding properties are similar to ferric ions, which may be bound to transferrin. So it can be considered as a competitive element to iron and can interfere with iron absorption. The aim of this study was to investigate the inhibitory effect of Cerium in presence of transferrin on gastric adenocarcinoma cells in vitro.

**Methods:** The adenocarcinoma cells were obtained from patients after a pathological confirmation, then they were cultured in DMEM environment and cytotoxic effect of different concentrations of cerium were measured (0.1, 1, 10 and 100 µM) in the presence and absence of transferrin, on periods 24 and 48 hours by MTT and LDH cytotoxic assay.

**Results:** The results of MTT and LDH measurements showed that Cerium itself has a cytotoxic effect on cancer cells isolated from the patient as well as it increases significantly in the presence of transferrin carrying a mortality rate of cancer cells (P <.05).

**Conclusion:** Cerium is competitive element in the mechanism of iron absorption and can interfere and inhibit the growth of adenocarcinoma cancer cells; also, the use of Cerium and transferrin simultaneously may cause a greater inhibitory effect.

## Introduction

According to the World Health Organization statistics, cancer is the second leading cause of deaths in developed countries and the third leading cause of death in developing countries [**5**]. Meanwhile, gastric cancer is particularly common among the gastrointestinal cancers. Gastric cancer is the fourth most common cancer in the world. The most common form, adenocarcinoma, or glandular cancer constitutes approximately 90% of stomach cancers and 10% constitute other types such as Lymphoma and Leiomyosarcoma. Therefore, it seems essential to research on creating mechanisms of this widespread cancer, as well as providing optimal therapies to combat it. Particularly, gastrointestinal cancers, especially gastric cancer, are a serious problem all over the world [**4**]. Compounds extracted from plants or plant extracts, had a long history in the treatment or prevention of cancer. However, in recent years, the study of minerals which has cytotoxic effects on tumor cells has been considered. For example, cytotoxic effects of lanthanides on various types of cancer cells and the action mechanism of these elements are subject of cytological experiments [**10**]. The subtle physiologic and normal interaction of these elements has not been clearly found with cells, but we know that cancer cells metabolize more iron than non-cancer and normal cells [**3**] Research on the transfer mechanism of lanthanide elements into cancer cells showed that the transfer of these elements and compounds like iron, transport into cells via receptor-mediated transport mechanism [**11**]. Furthermore, the other mechanisms, including the induction of necrosis and apoptosis is under investigation by these Elements [**6**,**7**]. The purpose of this study was to evaluate the anti-tumor effect Cerium (lanthanides) on the growth and survival of gastric adenocarcinoma cells isolated from patients in the presence of transferrin in vitro. 

## Method

Gastric adenocarcinoma cells isolated from the patients were centrifuged at 650 g o for 10 minutes, supernatant was discarded, and now residual cellular sediment in bottom of tubes containing blood cells and gastric adenocarcinoma, then 2 ml of Tyrode’s solution with 2.5 mM concentration was used to separate blood cells from gastric adenocarcinoma cells. After centrifugation for 13 min at 1000 g, gastric adenocarcinoma cells formed a white ring on the blood cell layer. The cells were gently collected by Pastor Pipet, then washed with DMEM containing 10% fetal bovine serum (FBS). To separate the cells, hyaluronidase enzyme was added and centrifuged for a few seconds; therefore, the medium was diluted and centrifuged again. Afterwards, supernatant discarded again and cells solved in 1 ml of the DMEM medium. Cell survival was determined by counting the number of colored cells with Trypan Blue dye and using hemocytomer LAM. Viable cells incubated in DMEM containing 10% fetal bovine serum, 1% penicillin amoxicillin streptomycin, 0.2% NaHCO3 and 1% 2 mM L-glutamine [**9**].

To test the effect of cerium, the 0.1, 1, 10 and 100 µL concentrations of Cerium sulfate were prepared, and then was filtered with 0.22 μ microbiological filters. The concentration of 20 μl of 100 μM sodium sulfate was used as a control. 180 μM of adenocarcinoma cell suspensions were used. The cells were plated in DMEM media and 96 well microplate at a concentration of 5×104 cell/ ml. 120 micrograms of transferrin were added in labeled rows, in wells that the cancer cells were cultured with 4 different concentrations of sulfate cerium. First, a cell suspension with a concentration of 5×104 cell/ ml was prepared in Falcon, and then it was exploited in 96-well plates. Samples were incubated at 37°C for 24 and 48 hours in an incubator containing 5% CO2 and 95% humidity. Using MTT assay, the percent of cell survival was measured by ELISA reader in 540 nm.

To investigate the anticancer effects of cerium on adenocarcinoma cells after sample preparation and adding different serial cerium concentrations in the presence and absence of transferrin and passing the time of incubation, the cerium induced cytotoxicity was determined by the MTT method [**8**].

In LDH assay, LDH activity released from damaged cells is measured, cytotoxic effects of different concentrations cerium sulfate in the presence of 120 mg of transferrin on the adenocarcinoma cell are assayed by colorimetric method. In MTT method, the tetrazolium salt 3 (4, 5-Thiazole dimethyl-2-yl)-2, 5-diphenyl tetrazolium bromide (MTT), which is a solution reduced by mitochondrial succinate Dehydrogenase of living cells forms indloublr purple Formosan which soluble in DMSO and measured by ELISA Reader in 540 nm, is used to calculate the cell survival. For the measurement of cytotoxicity, a simple and accurate colorimetric method for the determination of cytolysis, measures LDH activity released from damaged cells, Roche company lactate dehydrogenase assay kit, being used. The percentage of cell survival is calculated through the formula below:

Percentage of cell survival = (absorbance of test/ absorbance of control) ×100

**Statistics**

To investigate the Cerium cytotoxic effect on growth and survival of adenocarcinoma tumor cells isolated from patients, obtained data are expressed as mean ± SD. For the statistical significance, one-way ANOVA was used to analyze the differences between each sample and control (*P<0.05).

## Results

**Fig. 1:**
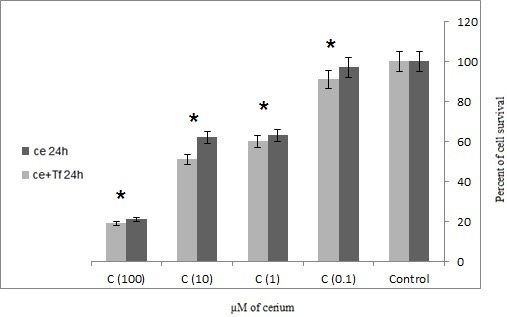
Effects of four concentrations alone or in the presence of serial Cerium transferrin on adenocarcinoma cells isolated from patients during 24 hours, MTT assay technique (Ce = Cerium, Tf = Transferrin) statistical significance one-way ANOVA was used to analyze the differences between each sample and control (*P<0.05)

**Fig. 2:**
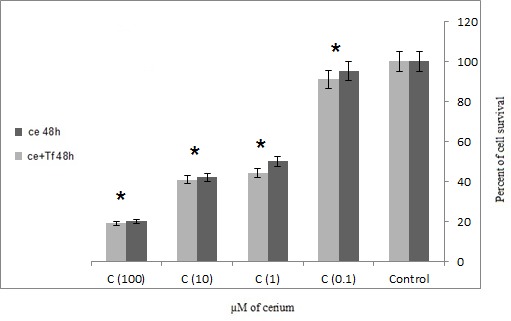
Effects of four concentrations alone or in the presence of serial Cerium transferrin on adenocarcinoma cells isolated from patients during 48 hours, MTT assay technique (Ce = Cerium, Tf = Transferrin) statistical significance one-way ANOVA was used to analyze the differences between each sample and control (*P<0.05)

MTT results showed that Cerium alone and in the presence of transferrin carries an increased significant mortality rate in adenocarcinoma cancer cells isolated from patients with gastric cancer (p=0.047). In the presence of transferrin, Cerium has a more significant effect on the mortality rate of cancer cells (**Fig. 1**,**2**).

The results showed that the 48 h incubation has the most significant effect on cancer mortality rate than 24 h (**Fig. 1**,**2**).

The results of LDH assay showed that cerium alone and in the presence of transferrin was significantly increased in cell survival of adenocarcinoma cells (p=0.001).

**Fig. 3:**
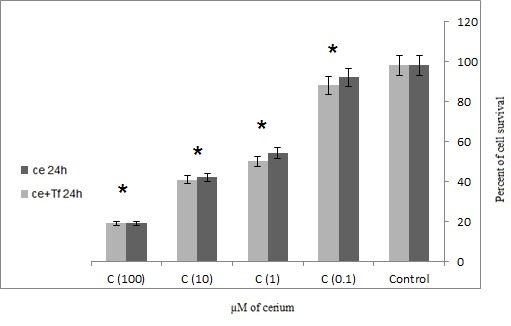
Effects of four concentrations alone or in the presence of serial Cerium transferrin adenocarcinoma cells which isolated from patients with LDH assay technique in 24 hours (Ce = Cerium, Tf = Transferrin) statistical significance one-way ANOVA was used to analyze the differences between each sample and control (*P<0.05)

**Fig. 4:**
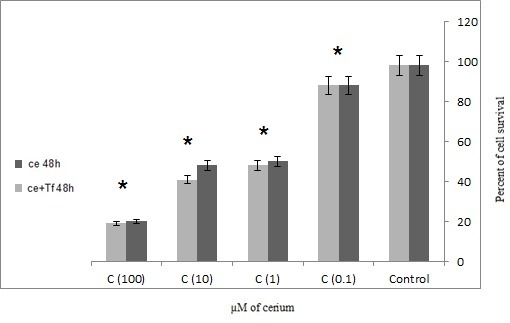
Effects of four concentrations alone or in the presence of serial Cerium transferrin adenocarcinoma cells which isolated from patients with LDH assay technique in 48 hours (Ce = Cerium, Tf = Transferrin) statistical significance one-way ANOVA was used to analyze the differences between each sample and control (*P<0.05)

Also, in the presence of transferrin Cerium has a more significant effect on the mortality rate of cancer cells (**Fig. 3**,**4**). The results showed that the 48 hours incubation of cells which were isolated from patients with gastric adenocarcinoma, had a more significant impact on cancer mortality to 24 hours incubation (**Fig. 3**,**4**).

**Pathology results:**

**Fig. 5:**
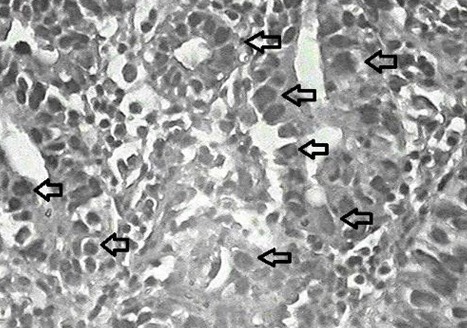
Pathologically confirmed adenocarcinoma cells isolated from patients (flashes are adenocarcinoma cells)

**Fig. 5** represents a pathology confirmed image of adenocarcinoma, gastric cancer cells shown by the arrows.

## Discussion

Transferrin is a protein carrier that transports iron in blood; iron is needed in many metabolic pathways in the cell especially required for the proliferation of cancer cells so these cells need more iron requirements, thus the expression of transferrin receptors is increased on the surface of cancer cells [**13**]. Increased Levels of transferrin receptor expression in cancer cells, such as epithelial cancer cells, cervical cancer cells, and breast cancer has been observed to provide more amount of available iron because iron is essential for cell proliferation and spread of cancer [**1**,**2**]. Thus, the principal roles of transferrin have been shown as a cell growth factor dependent on iron is crucial for the growth of cancer cells [**10**]. To study this phenomenon in isolated gastric adenocarcinoma, cells from patients were examined in the presence iron competitive element (Cerium) and its carrier Transferrin. 

The results are consistent with previous studies that transferrin increases cell proliferation in cancer cells. However, if the element is a competitive alternative to iron onto transferrin inhibits the growth of cancer cells. In this study, Cerium was used as a competitor of iron to inhibit tumor cell growth. **Fig. 1**,**2** showed that with or without the presence of the iron carrier transferrin, either 24 or 48 hour incubation with 0.1, 1, 10 and 100 µM of cerium can significantly inhibit tumor cell survival and it can propose the dose-dependent inhibitory effect of cerium. The same phenomenon was also observed in **Fig. 3**,**4** in which tumor cell death was measured after 24 and 48 hour incubation with cerium and transferrin by a LDH assay.

Therefore, this phenomenon can introduce cerium as a candidate to inhibit cancer cell proliferation [**12**] and the effectiveness of cerium in the presence of transferrin showed that this element may have a similar mechanism of endocytosis mediated by transferrin receptor [**12**]. Due to the similarity of the chemical properties of cerium with iron ions, cancer cells generally express much higher transferrin receptors than the normal cells. Therefore, they may inhibit the malignant cell type’s dependent on metal ion with such substances and mechanisms.

In conclusion, this study can describe cerium and transferrin effect on cell growth inhibition in adenocarcinoma cells, but the actual mechanism of action remains unclear, which will need further studies to be understood.

**Acknowledgments**

The cost of this study was provided by the Research Council of Lorestan University of Medical Sciences. 

## References

[1] Aisen P (1998). Transferrin, the transferrin receptor, and the uptake of iron by cells. Metal Ions Biol Syst.

[2] Baker HM, Baker CJ, Smith CA, Baker EN (2000). Metal substitution in transferrins: specific binding of cerium (IV) revealed by the crystal structure of cerium-substituted human lactoferrin. J Biol Inorg Chem.

[3] Dai Y, Li J, Li J (2002). Effects of rare earth compounds on growth and apoptosis of leukemic cell lines. In Vitro Cell Dev Biol Anim.

[4] Hiroyuki O (2006). Early gastric cancer: diagnosis, pathology, treatment techniques and treatment outcomes. European Journal of Gastroenterology & Hepatology.

[5] Jemal A, Murry I, Ward E, Samuels A, Tiwari RC, Chafoor A, Feuer EJ, Thun MJ (2005). Cancer Statistics. CA Cancer J Clin.

[6] Ji Yj, Xiao B, Wang ZH (2000). The suppression effect of light rare earth elements on proliferation of two cancer cell lines. Biomed Environ Sci.

[7] Jiang XP, Wang F (2002). Induction of apoptosis by iron depletion in the human breast cancer MCF-7 cell line and the 13762NF rat mammary adenocarcinoma in vivo. Anticancer research.

[8] Mosmann T (1983). Rapid colorimetric assay for cellular growth and survival: Application to proliferation and cytotoxicity assays. J. Immunol. Methods.

[9] Mitruţ P, Burada F, Enescu A (2009). The genotoxicity study of resveratrol in primary gastric adenocarcinoma cell cultures. Rom J Morphol Embryol.

[10] Palizban AA, Sadeghi-Aliabadi H, Abdollahpour F (2010). Effect of cerium lanthanide on Hela and MCF-7 cancer cell growth in the presence of transferring. Res Pharm S.

[11] Yang B, Feng J (2002). The Functions of transferrin and its receptor with lanthanides and other metal ions. Progress in Chemistry.

[12] Zende-Del A, Ahmadvand H, Abdollah-Pour F, Abdolahian M, Ahmadi-Nejad M, Alie-Poor A, Safari M (2013). Cerium lanthanide effect on growth of AGS cell line with the presence of transferrin in vitro. Cerium lanthanide effect on growth of AGS cell line with the presence of transferrin in vitro.

[13] Zhang M, Gumerov DR, Kaltashov IA, Mason AB (2004). Indirect detection of protein-metal binding: interaction of serum transferrin with In3+ and Bi3+. J Am Soc Mass Spectrom.

